# Genome-wide characterization and analysis of rice *DUF247* gene family

**DOI:** 10.1186/s12864-024-10515-8

**Published:** 2024-06-18

**Authors:** Feifei Zhang, Yixi Liu, Fang Liu, Jun Yang, Amir Sohail, Chengkai Lu, Peng Xu

**Affiliations:** 1 Key Laboratory of Tropical Plant Resources and Sustainable Use, Xishuangbanna Tropical Botanical Garden, Chinese Academy of Sciences, Kunming, 650223 China; 2 University of Chinese Academy of Sciences, Beijing, 100049 China; 3 Biotechnology and Germplasm Resources Institute, Yunnan Academy of Agricultural Sciences, Kunming, 650205 China; 4 The first affiliated hospital of Kunming Medical University, Kunming, 650032 China

**Keywords:** *DUF247* gene family, Gene characterization, Phylogenetic analysis, Expression pattern, Abiotic stress, Rice

## Abstract

**Background:**

The domain of unknown function 247 (DUF247) proteins is involved in plant development and stress response. Rice is an important worldwide cereal crop, although an increasing number of DUF proteins have been identified, the understanding of DUF proteins is still very limited in rice.

**Results:**

In this study, we identified 69 genes that encode DUF247 proteins in the rice (*Oryza sativa*) genome by homology searches and domain prediction. All the OsDUF247 proteins were classified into four major groups (I, II, III and IV) by phylogenetic analysis. Remarkably, *OsDUF247* genes clustered on the chromosomes solely show close phylogenetic relationships, suggesting that gene duplications have driven the expansion of the *DUF247* gene family in the rice genome. Tissue profile analysis showed that most *DUF247* genes expressed at constitutive levels in seedlings, roots, stems, and leaves, except for seven genes (*LOC_Os01g21670*, *LOC_Os03g19700*, *LOC_Os05g04060*, *LOC_Os08g26820*, *LOC_Os08g26840*, *LOC_Os08g26850* and *LOC_Os09g13410*) in panicles. These seven genes were induced by various abiotic stress, including cold, drought, heat, hormone treatment, and especially salt, as demonstrated by further experimental analysis. DUF247 proteins contain transmembrane domains located on the membrane, suggesting their significant roles in rice development and adaptation to the environment.

**Conclusions:**

These findings lay the foundation for functional characterizations of *DUF247* genes to unravel their exact role in rice cultivars.

**Supplementary Information:**

The online version contains supplementary material available at 10.1186/s12864-024-10515-8.

## Introduction

The domain of unknown function (DUF) is a conserved protein domain whose function remains unknown. DUF proteins usually contain one or more conserved DUF domains and are classified into different families based on the type of DUF domain [1]. The availability of large amounts of transcriptome and genomic sequence data has led to the identification of numerous DUF proteins. Currently, there are approximately 5889 entries of DUF proteins in the Pfam database [2].

Numerous DUF genes have been studied in different plant species. Previous studies have suggested that DUF proteins are widely involved in various biological processes [3, 4]. Some of them play key roles in plant growth and development. In *Arabidopsis*, *DUF640* and *DUF724* have been demonstrated to be involved in seedling development, cell growth, and chloroplast movement [5]. Members of the DUF724 protein family have been found to regulate cell polarity growth in the apical meristem of buds, leaves, and roots [6]. A pair of DUF538 domain-containing proteins was shown to modulate plant growth and trichome development [7–9]. Moreover, TH1/BSG1 which belongs to the DUF640 family is a positive regulator of flower development by controlling inner and outer ridge development [10]. However, *OsRMC* from the *DUF26* gene family negatively regulates root elongation, root numbers, and curliness in rice [11].

In addition to involvement in plant growth and development, DUF proteins have been implicated in stress response as well. The *DUF26* has been demonstrated to have a positive regulatory role in plant defense against *Ustilago maydis* in maize and *Pseudomonas syringae* in *Arabidopsis* [12, 13]. On the other hand, *AtDUF569* has been shown to have a negative regulatory effect on Arabidopsis resistance to *Pseudomonas syringae pv. tomato* DC3000 by upregulating the expression of SA-dependent PR genes. The suppression of Arabidopsis RING-DUF1117 E3 ubiquitin ligases, AtRDUF1 and AtRDUF2, reduced tolerance to ABA-mediated drought stress [14]. Overexpression of *DUF966* in rice promoted seedling tolerance to high salinity and PEG6000 stress [15]. Expression profiling of *ATDUF4228* genes under abiotic stresses such as osmotic, salt, and cold stress suggested that some *ATDUF4228* genes may be involved in plant resistance pathways against abiotic stresses [16]. Furthermore, *StDDP5* and *StDDP7*, both of which belong to the *DUF221* gene family, exhibited high expression against heat and salt stress, respectively, in potatoes [17].

Several reports have analyzed the structure of the DUF family. In tobacco, Motifs 1 and 7 were consistently found in all DUF868 proteins [18]. In *Zingiber officinale*, Motif 1, Motif 2, and Motif 4 demonstrated significant conservation across the evolution of 12 ZoDUF668 genes [19]. Amone 10 conserved motifs, Motif 1 was universally present in all 489 DUF proteins, while Motif 3, Motif 4, and Motif 6 were prevalent among most DUF4228 proteins in land plants [16]. Additionally, Motifs 1, 4, and 5 were identified in all OsDUF568s in rice [20]. The *DUF4228* genes from angiosperms formed a distinct cluster, separate from other plant lineages. Additionally, *DUF4228* genes from gymnosperms clustered together on a large branch alongside those from angiosperms. Within angiosperms, *DUF4228* genes from monocots and dicots exhibited clear separation on different branches [16]. Furthermore, the genetic distance of *DUF568* between rice and maize is relatively close, while genes in *Arabidopsis* were comparatively distant and displayed higher conservation [20].

Although certain DUF gene families have been elucidated, a significant portion of the DUF members remain obscure, particularly in the case of rice. The *DUF247* gene family is a large gene family that encode proteins containing a conserved domain with amino acids ranging from 67 to 711 [2, 21–24]. This gene family is widely present in monocot and dicot plant species, indicating its importance. In *Arabidopsis*, one member of the *DUF247* gene family, *DLE1*, was found to be a positive regulator of plant defense response [21]. The DUF247 domain-containing *LpSDUF247* which protein is considered to be the S-locus male determinant in the gametophytic SI system is highly expressed in pollen and stigma but has low expression in vegetative organs in perennial Ryegrass [22]. Similarly, *OlSS1* and *OlSS2*, which are homologous to *LpSDUF247*, are syntenic with *OsDUF247* (*Os05g0197900*) and have been shown to be involved in gametophytic self-compatibility in *O. longistaminata* [23]. Moreover, DUF247 proteins have also been implicated in sex determination in garden asparagus [25, 26]. Recently, the *DUF247* gene family has been identified as one of the largest and most densely tandemly duplicated gene families in the oak genome (*Quercus lobata Née*, valley oak; tree SW786) [24]. These findings imply that *DUF247* genes play pivotal roles in fundamental biological processes in plants, despite the lack of characterization or functional investigation specifically within the context of rice.

In this study, the predicted DUF247-encoding genes were identified in the rice genome and a phylogenetic analysis was conducted. Furthermore, their gene structures, phylogenetic relationships, expression profiles under various abiotic stresses, and protein subcellular localizations were also examined. The studies indicate important functional characterizations of the *DUF247* gene family and can be a base and guide for research into the *DUF247* gene family in rice.

## Materials and methods

### The identification of rice *DUF247* genes

To identify members of the *DUF247* gene family in plants, a hidden Markov model (HMM) of the DUF247 (Pfam PF03140) domain was obtained from Pfam 31.0. The Hmmsearch tool from the HMMer package version 3.1b1 was employed to search the DUF247.hmm against the protein sequences from each plant genome (https://phytozome.jgi.doe.gov). To ensure search reliability, domain hits beyond the gathering threshold (E-value 1e^− 10^) were filtered out before downstream analysis. In cases where gene loci had multiple predicted isoforms, the primary isoform was selected if annotation was available; otherwise, the longest protein was chosen. The remaining sequences were manually checked for the conserved DUF247 domain using Pfam and SMART (http://smart.embl-heigelberg.de/) [27]. Additionally, to further ensure search reliability, domain hits with an E-value greater than 1e^− 10^ and those with less than 80% coverage were manually filtered out before downstream analysis.

### Sequence analysis

To better understand the properties of OsDUF247 proteins, we uploaded all protein sequences to ExPASy for calculation of their molecular weights and isoelectric points. Additionally, subcellular localizations were predicted using the Plant-Ploc server (http://www.csbio.sjtu.edu.cn/bioinf/plant-multi/).

### Phylogenetic analysis

The *DUF247* genes were aligned by Muscle (fast) in codon with FasParser v2.10.0 [28]. Poorly aligned regions were trimmed using GBLOCKS v.0.91b with relaxed settings [29]. The un-rooted phylogenetic tree inferred by the neighbor-joining (NJ) method using MEGAX with the following parameters: Poisson model, partial deletion, and 1000 bootstrap replications [30].

### Gene structure and conserved motifs characterization

The amino acid sequences of OsDUF247 proteins in rice were compared using the MUSCLE method in MEGAX and visualized using Jalview. The software TBtools was used to examine the exon-intron structures of rice *OsDUF247* genes [31]. Conserved motifs were predicted by the MEME program (http://meme-suite.org/tools/meme).

### Chromosomal location, gene distribution and synteny analysis

*OsDUF247* genes were mapped to rice chromosomes based on the retrieved annotation file of rice genome (*Osativa*_323_v7.0). Gene duplication events were analyzed by using Multiple Collinearity Scan toolkit (MCScanX) with the default parameters [32]. To visualize the syntenic relationship of *OsDUF247* genes in the rice genome, the syntenic analysis maps were constructed using the MCScanX.

### Plant materials and abiotic stress treatments

Two typical rice varieties, Minghui63 (*Indica*) and Nipponbare (*japonica*), were used throughout the experiment. The rice plants were grown in an artificial climate chamber under a photoperiod of 16 h light/8 hours dark at 28℃. The three-week-old seedlings were transferred to containers with 1/2 MS liquid medium and maintained under the same conditions for one week before abiotic stress treatment, which was carried out as previously described [33]. For phytohormone treatments, roots of seedlings were submerged for 24 h to liquid 1/2 MS media without and with gibberellin (GA_3_, 50 µM), abscisic acid (ABA, 100 µM). The other abiotic stresses were: four-week-old seedlings were subjected to cold (4℃ for 12 h), drought (23% PEG 6000 solution for 12 h at 28℃), heat (42℃ for 2 h and recovery at 28℃ for 24 h) and salt (200 mM NaCl for 12 h at 28℃) [33]. Tissue samples including root, leaf, stem, panicle, stamen, pollen, and endosperm from different developmental stages were collected separately for RNA extraction and subsequent qRT-PCR analysis. To investigate the expression patterns of selected genes under salt stress, four-week-old seedlings were subjected to a 200 mM NaCl solution for varying durations of 2, 4, 7, 18, and 48 h.

### RNA extraction and qRT-PCR analysis

Total RNA was extracted using Trizol reagent (Invitrogen, Carlsbad, CA, USA), and the concentration of each RNA sample was quantified using a NanoDrop ND-1000 spectrophotometer. First-strand cDNA synthesis was performed using the HiScript II 1st Strand cDNA Synthesis kit (Vazyme) following the manufacturer’s recommendations. Quantitative real-time PCR (qRT-PCR) was conducted on a Roche Lightcycler 480 II instrument with SYBR Green chemistry. The rice actin gene was used as an internal control. The reaction procedure was as follows: initial denaturation at 95℃, for 30 s, followed by 40 cycles of denaturation at 95℃ for 10 s, annealing at 60℃ for 30 s, and extension at 72℃ for 30 s. A final extension step was conducted at 72℃ for 5 min. Afterward, the samples were held at 12℃ indefinitely. Each reaction was conducted in triplicate, and the data from qRT-PCR were analyzed using the 2^−ΔCT^ method. The primer sequences used are listed in Supplementary Table 1.

### Expression analysis of *OsDUF247* under abiotic stress

The expression pattern of *DUF247* genes in the seedling, root, stem, leaf, young panicle, and stamen tissues of the *indica* cultivar Minghui63 under normal and stress conditions were obtained from the public expression database CREP [34] (http://crep.ncpgr.cn/), which is a public expression database from the Affymetrix GeneChip Rice Genome Array. There were three biological replicates for each treatment. Additionally, three microarray databases were used to analyze the expression of *DUF247* genes under salt stress conditions: GSE6901 for the *indica* IR64 (http://www.ncbi.nlm.nih.gov/geo/query/acc.cgi?acc¼GSE6901) [35, 36], GSE3503 for the *indica* IR29 (http://www.ncbi.nlm.nih.gov/geo/query/acc.cgi?acc¼GSE3053) [37], and GSE4438 for *indica* IR29, MH63, and *japonica* M103 (http://www.ncbi.nlm.nih.gov/geo/query/acc.cgi?acc¼GSE4438) [38]. To identify the tissues where a gene was specifically expressed, the log_2_-transformed expression values of the gene across all organs were ranked. The largest difference (max gap) of expression values between adjacent tissues was utilized as the cutoff for differential expression. Tissues preceding the largest gap were considered as where the gene was specifically expressed. Consequently, expression values of the genes in these tissues would be at least 2^max gap^ fold higher than in other tissues.

The original expression data for *OsDUF247* genes under various treatments, including gibberellins (GA, PRJNA408068), paclobutrazol (PB, PRJNA408068), salt stress (PRJEB4672), and drought stress (PRJNA272723) were retrieved from the NCBI SRA (Available online: https://www.ncbi.nlm.nih.gov/). RNA-seq data were downloaded from the NCBI database and mapped to genomes using Hisat2 (version 2.2.1, default settings) [32]. The expression level (Fragments Per Kilobase of exon model per million mapped fragments) was calculated by Cufflinks. BLAST was used to compare DNA or protein sequences against genomic or transcriptomic databases to confirm that the *DUF247* genes in all the species we used was not a pseudogene. The default parameters were adopted and the cutoff value was set to 0.01. A heat-map was generated using Amazing Heat Map software within the TBtools suite [39] with average linkage hierarchical clustering to show the differential expression levels of *OsDUF247* genes across the different treatments.

## Results

### Phylogenetic analysis of DUF247 families in rice, other Gramineae species, and dicots

A total of 179 protein sequence entries with a predicted DUF247 domain were found after searching the public database through NCBI and Phytozome website with domain number PF03140. To eliminate redundancies, sequences encoding the same proteins and non-representative transcripts were excluded from the analysis. The remaining protein sequences were then screened to ensure the presence of a complete DUF247 domain by HMMER, SMART, CDD, and Pfam. After removing non-DUF247 domain proteins and those with estimated E-values above 1 × 10^− 10^, we identified 69 non-redundant *DUF247* genes in the rice genome (MSU 7.0). Detailed information regarding the identified DUF247 domains is provided in Supplementary Table 2.

The length of rice DUF247 proteins ranges from 75 (LOC_Os06g08760) to 711 (LOC_Os02g15430) amino acids. The conserved DUF247 domain typically contains about 66–455 amino acids, accounting for the divergent length of the *DUF247* genes. The molecular weight of rice DUF247 proteins ranges from 8.68 kDa (LOC_Os06g08760) to 80.03 kDa (LOC_Os02g15430). The *DUF247* genes are randomly distributed across 12 chromosomes, except for one gene (pentatricopeptide, *PPR*), which could not be anchored onto any chromosome. The predicted isoelectric points of these proteins range from 5.03 (LOC_Os08g42570) to 11.2 (LOC_Os07g47520). A total of 1,439 pseudogenes were identified with pseudo gene models, and 12% of these genes were located in intergenic regions, lacking definable open reading frames. The presence of the *PPR* gene within an unanchored contig in the rice genome may be attributed to the inherent complexity of plant genomes, including those of rice. This may explain why the *PPR* gene could not be anchored onto any chromosome. Information about *DUF247* gene family members including the positions on the chromosomes, the predicted subcellular localizations were presented in Supplementary Table 3.

**Fig. 1:**
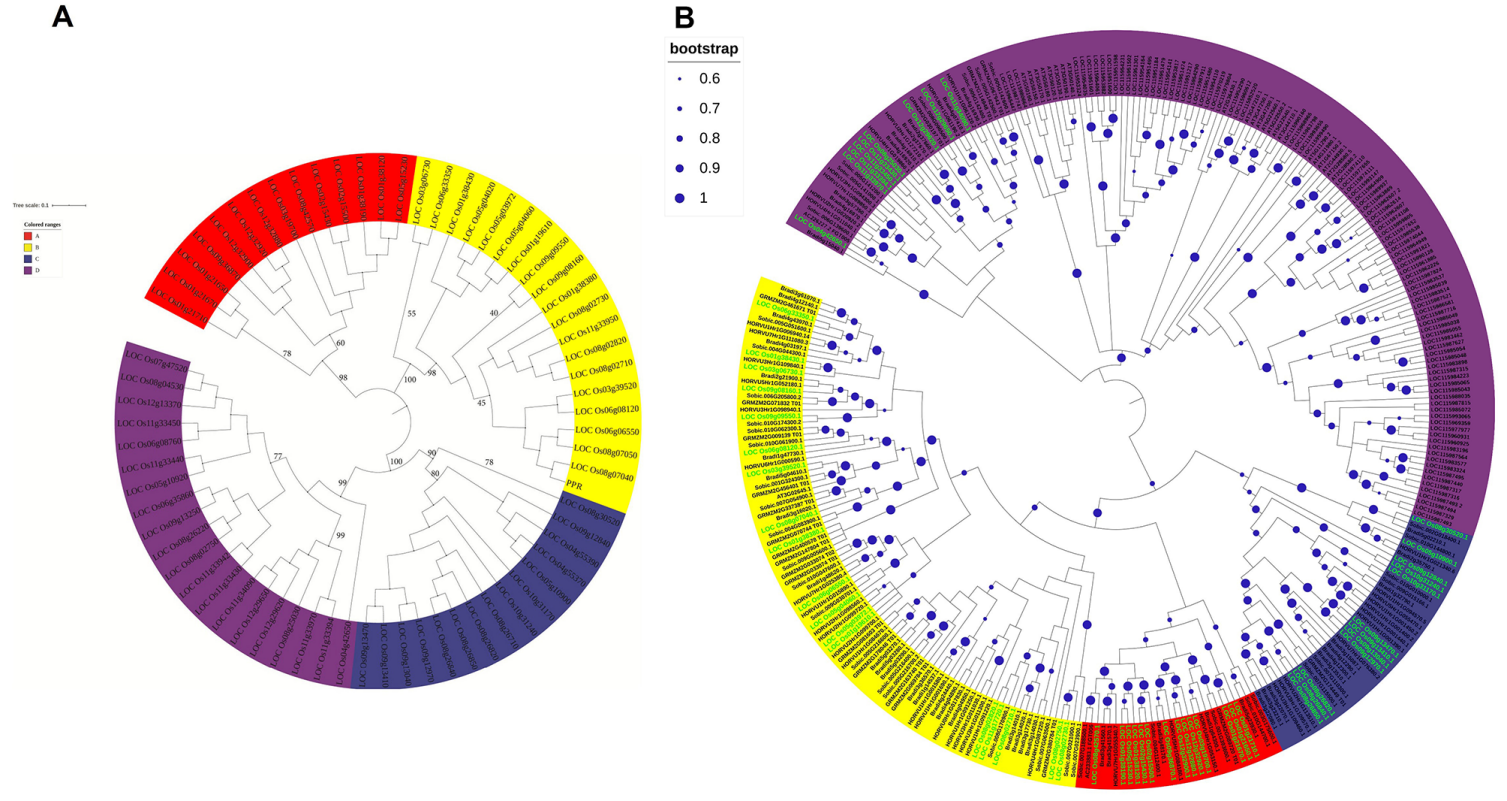
Phylogenetic analysis of DUF247 families. **A**, Phylogenetic relationships of the identified 69 *DUF247* genes in rice. **B**, Phylogenetic analysis of the *DUF247* genes from rice (LOC_Os), brachypodium (Bradi), sorghum (Sobic.), maize (GRMZM2G), barley (HORVU), Arabidopsis (AT), and oak (LOC). An unrooted phylogenetic tree depicting the relationships among DUF247 domains in rice was constructed by aligning DUF247 domain sequences using software MEGAX with 1000 bootstrap. Tree’s colored groups represent different groups or sub-groups of the DUF247 domain. In these five Gramineae (rice, brachypodium, sorghum, maize, and barley) and two dicots (Arabidopsis and oak), the DUF247 family is classified into four distinct classes, as illustrated in Fig. 1A. The green highlight represents the rice members of the DUF247 family

To investigate the evolutionary relationships among DUF247 members in rice, we used 69 rice *DUF247* genes to construct rooted phylogenetic tree using MEGAX with a bootstrap of 1000. The resulting phylogenetic tree (Fig. 1A) showed that the 69 *DUF247* genes were classified into four clades, representing distinct phylogenetic lineages, each supported by a bootstrap value over 80%. Group A and C contained 14 and 15 members, respectively, while groups B and D contained 20 members each. Some genes located on the same chromosomes clustered together in the phylogenetic tree, indicating that tandem duplication events may have occurred on the same chromosome.

To investigate the conservation of *DUF247* gene family members among Gramineae species and dicots, we utilized Pfam (PF03140) and HMMER to search the whole genomes of various Gramineae species (brachypodium, sorghum, maize, and barley) as well as dicots (Arabidopsis and oak). The numbers of *DUF247* genes used were 45, 37, 22, and 42 in Gramineae species, and 23 and 94 in dicots, respectively. Non-redundant *DUF247* genes were identified in their respective genomes (Supplementary Table 4). Phylogenetic analysis delineated the integration of DUF247 proteins into four clades (Fig. 1B). Notably, Class II harbors a greater abundance of genes from each species. Dicotyledonous plants are grouped into a single branch, forming a sister branch with Gramineae plants, (Fig. 1B), indicating a shared common ancestor.

### Gene structure, conserved domain and motif analysis of *DUF247* gene family

**Fig. 2:**
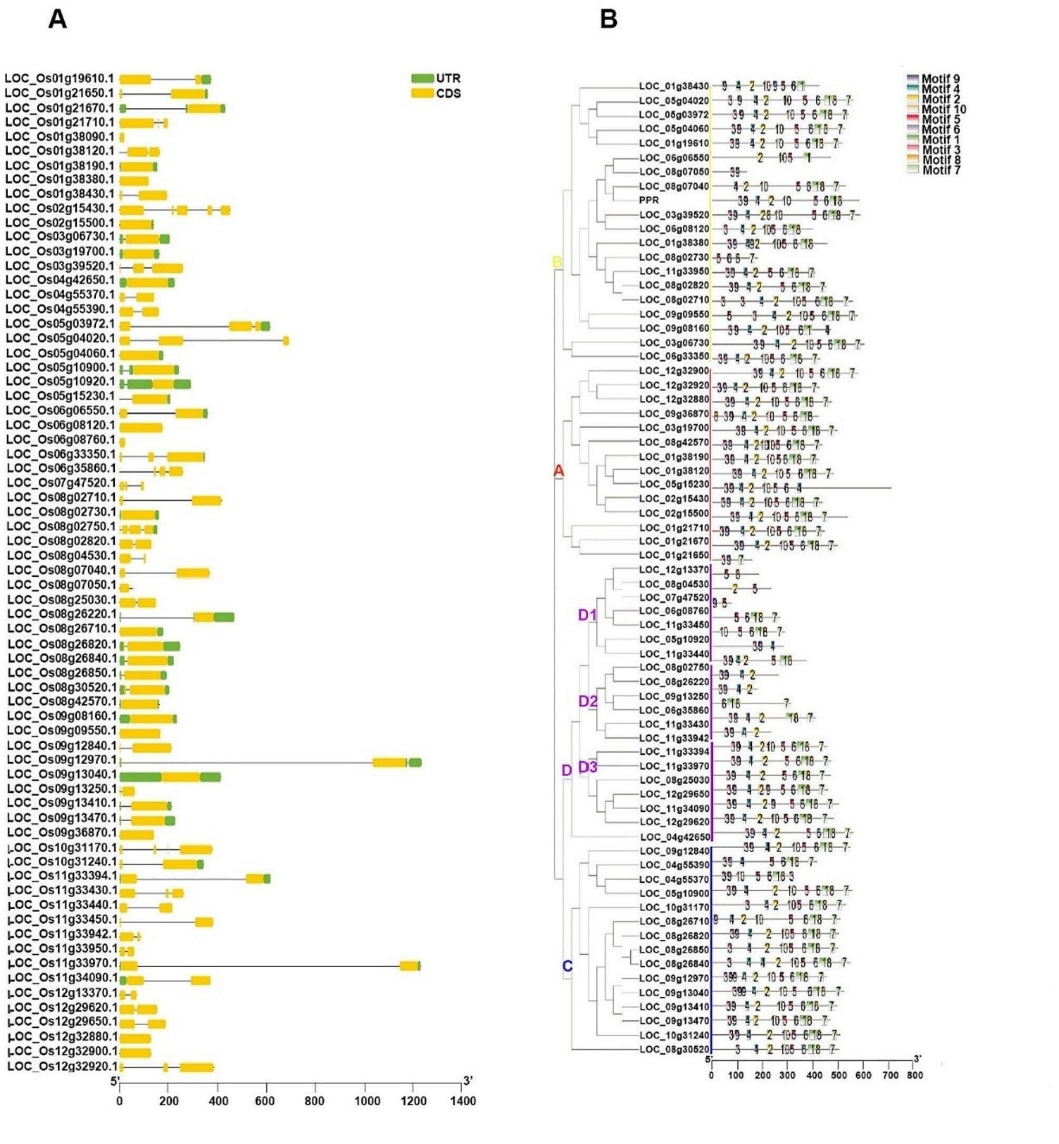
Gene structure and motif analysis of *DUF247* gene family. **A**, Gene structure of rice *DUF247* family. In the given representation, the green blocks represent the untranslated region (UTR), the yellow blocks represent exons represent introns. **B**, Phylogenetic relationships and architecture of conserved *DUF247* genes from rice were analyzed. Phylogenetic tree was constructed using MEGAX software, based on the full-length sequences of rice DUF247 proteins. In the tree, different colored boxes represent 10 predicted motifs, and the motif sizes are indicated by a scale. For more details about the motifs, please refer to Supplementary Table S5

Given the importance of gene organization in the evolution of gene families (Xu et al., 2012), we determined the gene structures and phases of introns/exons in *DUF247* genes by aligning genomic DNA and full-length cDNA sequences. The number of introns varied from 0 to 4 among the *DUF247* genes, with 18 (26%) genes lacking introns, 36 (52%) genes containing only one intron, and more than half of the members having more than one intron (Fig. 2A). In plants, the number of introns has been shown to be related to gene expression levels, with more introns generally associated with higher expression levels. However, compact genes may lead to rapid expression when exposed to environmental conditions.

We identified ten motifs in the conserved domains of DUF247 proteins with the MEME program, (Supplementary Table 5). Each protein contained a varying number of conserved motifs, ranging from 2 to 11, and the arrangement of motifs in genes within the same clade was almost identical. Each motif except for motif 10 was found only once in each DUF247 protein sequence. Most members of Clade A shared all ten motifs, with an arranged motif pattern of 3-9-4-2-10-5-6-1-8-7. Some members of Clades B and C shared double “9” motifs. Clade D could be further divided into three sub-groups based on their motif patterns and numbers (Fig. 2B). Sub-group D1 had the fewest number of motifs, while sub-group D2 had an incomplete motif pattern of 3-9-4-2-10-5-6-1-8-7, and sub-group D3 had an almost complete motif pattern, except for motif 10. Furthermore, gene domain analysis revealed a consistent pattern, that is, genes within the same group exhibited comparable domain quantities and lengths. Specifically, all members of the *DUF247* gene family possessed a solitary domain, while variations in domain length were observed among genes within the same cluster, aligning seamlessly with their corresponding phylogenetic relationships.

As observed through gene domain analysis, members within the same group of *DUF247* genes shared similar domain numbers and lengths. All *DUF247* gene members were found to have only one domain, and the length of these domains varied among genes within the same cluster, consistent with their phylogenetic relationships (Supplementary Table 2).

In general, closely related DUF247 proteins in adjacent clades or sub-clades of the phylogenetic tree had the same or similar motif structures. The extensive sequence diversity observed in the conserved domain suggests that domain shuffling after genome duplication may have occurred (Morgenstern and Atchley, 1999). However, as the domain range in this gene family is extensive, almost all motifs were included in the domain. This may explain why the motif structure among the four sub-groups was so similar, indicating that no domain shuffling occurred in the structure of the DUF247 protein family.

### Chromosome location and syntenic analysis of *DUF247* genes

**Fig. 3:**
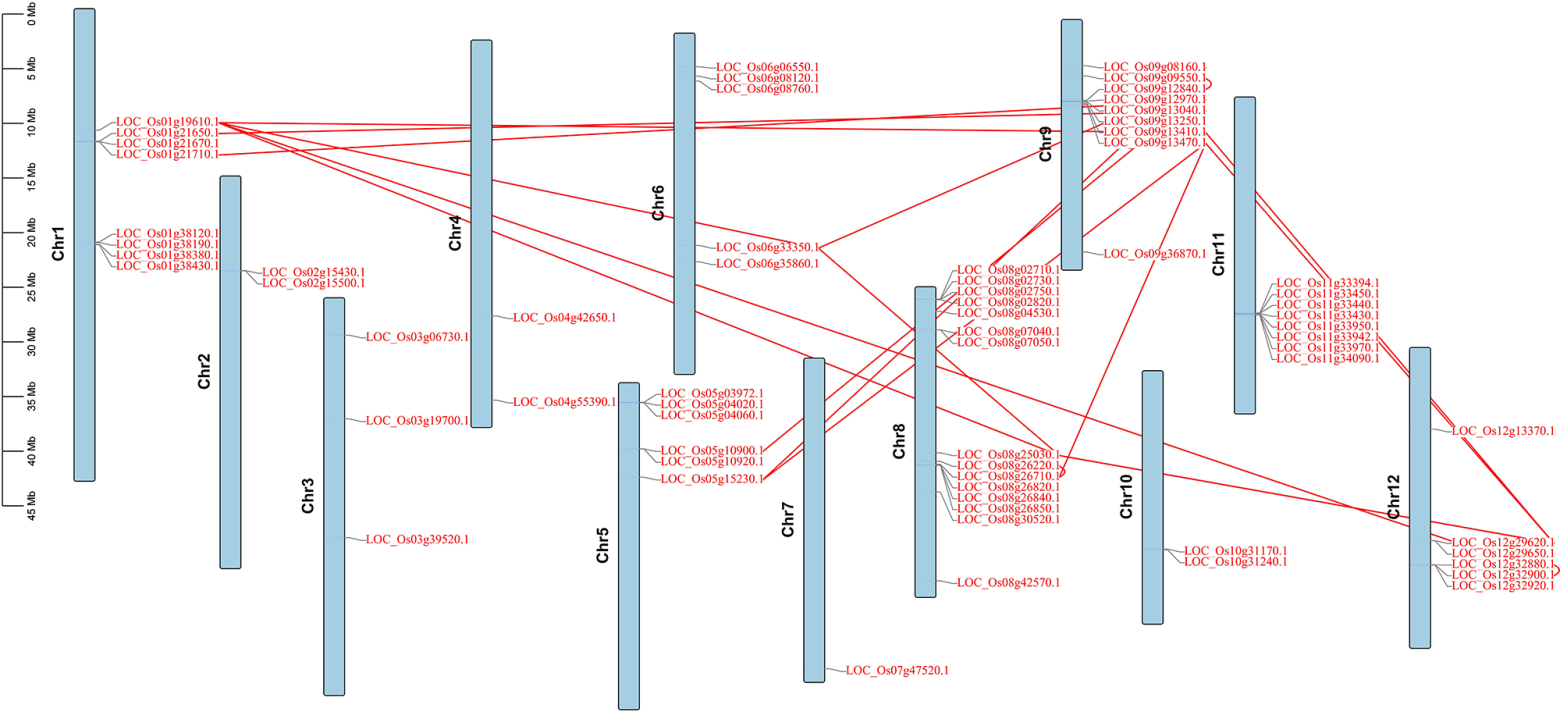
Chromosome distribution and gene duplications of *OsDUF247* genes. Tandem duplicated genes are represented by red arcs linking them together, while the red straight lines indicate segmental links between genes. The scale bar on the left side of the figure indicates the length (bp) of rice chromosomes

The distribution of *DUF247* genes on the chromosomes was uneven, with varying numbers of genes across chromosomes (Fig. 3). The number of *DUF247* genes on each chromosome ranged from 1 to 15. Chromosome 8 contained the most *DUF247* genes, with a total of 15, while only one gene was located on chromosome 7. These *DUF247* genes were observed to be distributed on both the distal and proximal ends of chromosomes.

**Fig. 4:**
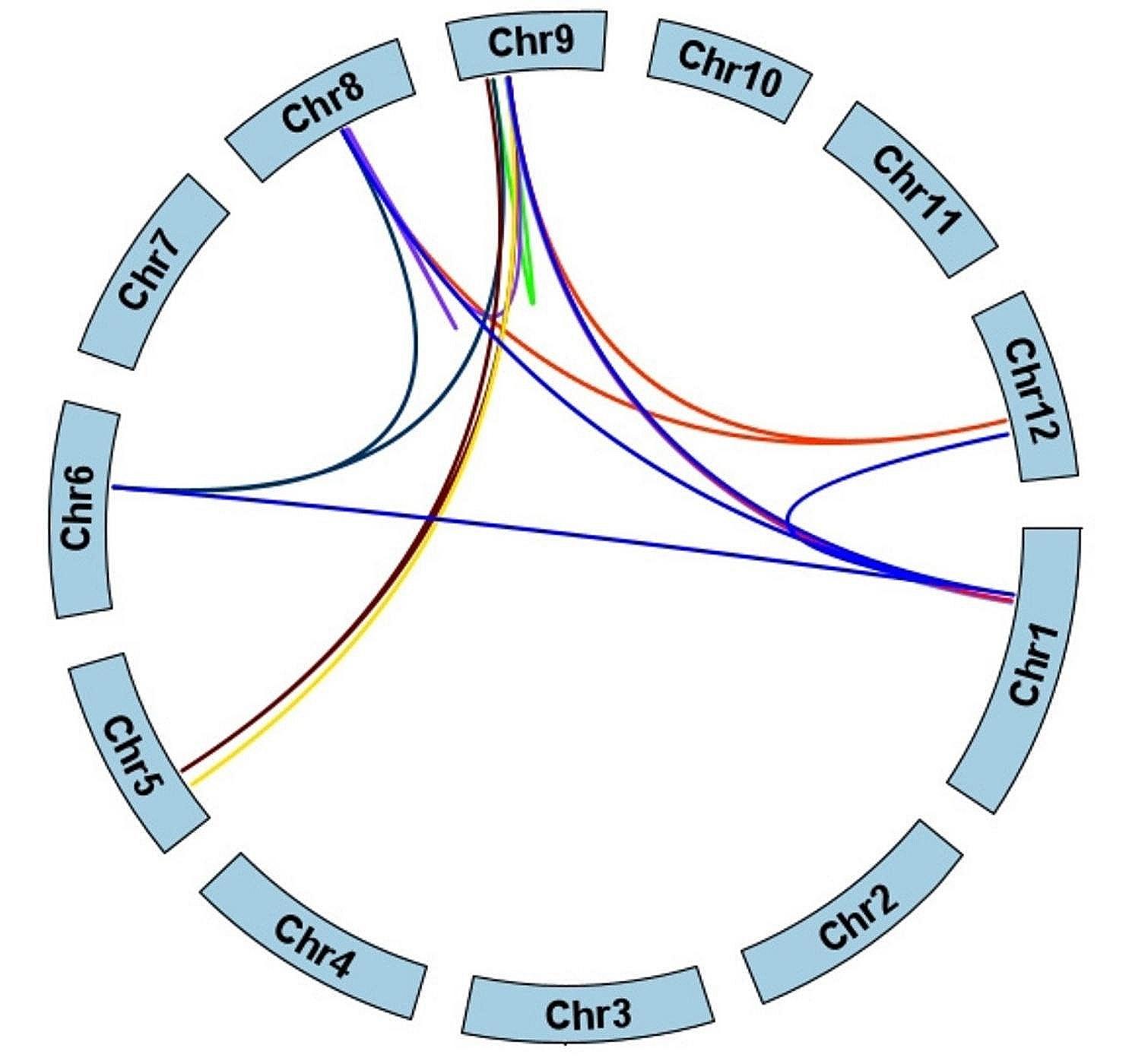
The inter-chromosomal relationships of *DUF247* genes. The colored lines represent segmental duplicated *DUF247* gene pairs. The chromosome number is indicated at the bottom of each chromosome

Gene duplication plays a crucial role in the evolution of plants. Gene family expansion and genomic evolutionary mechanisms partly depend on gene duplication events. In this study, we identified gene duplication events in the *DUF247* gene family. *DUF247* gene pairs resulting from segmental and tandem duplications were marked with red lines and red arcs, respectively. As shown in Fig. 3, three tandem duplications were found on chromosomes 8, 11, and 12, accounting for about 10% of the gene family, and were indicated by bending lines. Gene pairs such as *LOC_Os08g26220*-*LOC_Os08g26710* and *LOC_Os09g09550*-*LOC_Os09g12840* have been duplicated several times to form more than one gene pair with the other genes. The duplication on chromosome 12 was too close to resolve their duplication order (detailed duplication information is listed in Supplementary Table 6). Subsequent synteny analysis of the *DUF247* gene family revealed an extensive occurrence of over 18 segmental duplication events (Fig. 4). Notably, these duplications transpired not only within genes residing on the same chromosomes but also across different segments of the chromosomes, implying that duplication events likely served as the primary mechanism driving the expansion of the *DUF247* gene family in rice. Among the identified duplicated gene pairs, there were a total of 18 segmental duplicates (86%) and 3 tandem duplicates (14%). Remarkably, within the segmental duplicates, 7 of the 18 pairs (39%) belonged to the same phylogenetic clusters. Similarly, 1 out of the 3 tandem duplicates (33%) clustered together phylogenetically. For the remaining gene pairs, close proximity on the phylogenetic tree indicated substantial similarities in their protein domains.

To gain more insight into the evolutionary constraints acting on the *DUF247* gene family, we calculated the Ka/Ks ratio of *DUF247* gene pairs. Most segmental and tandem duplicated *DUF247* gene pairs had a *Ka/Ks* ratio < 1 (86%), indicating that the rice *DUF247* gene family likely experienced strong purifying selective pressure during evolution. Only three gene pairs had a *Ka/Ks* ratio > 1, suggesting that positive selection also played a role in the evolution of the *DUF247* gene family (Supplementary Table 6).

**Fig. 5:**
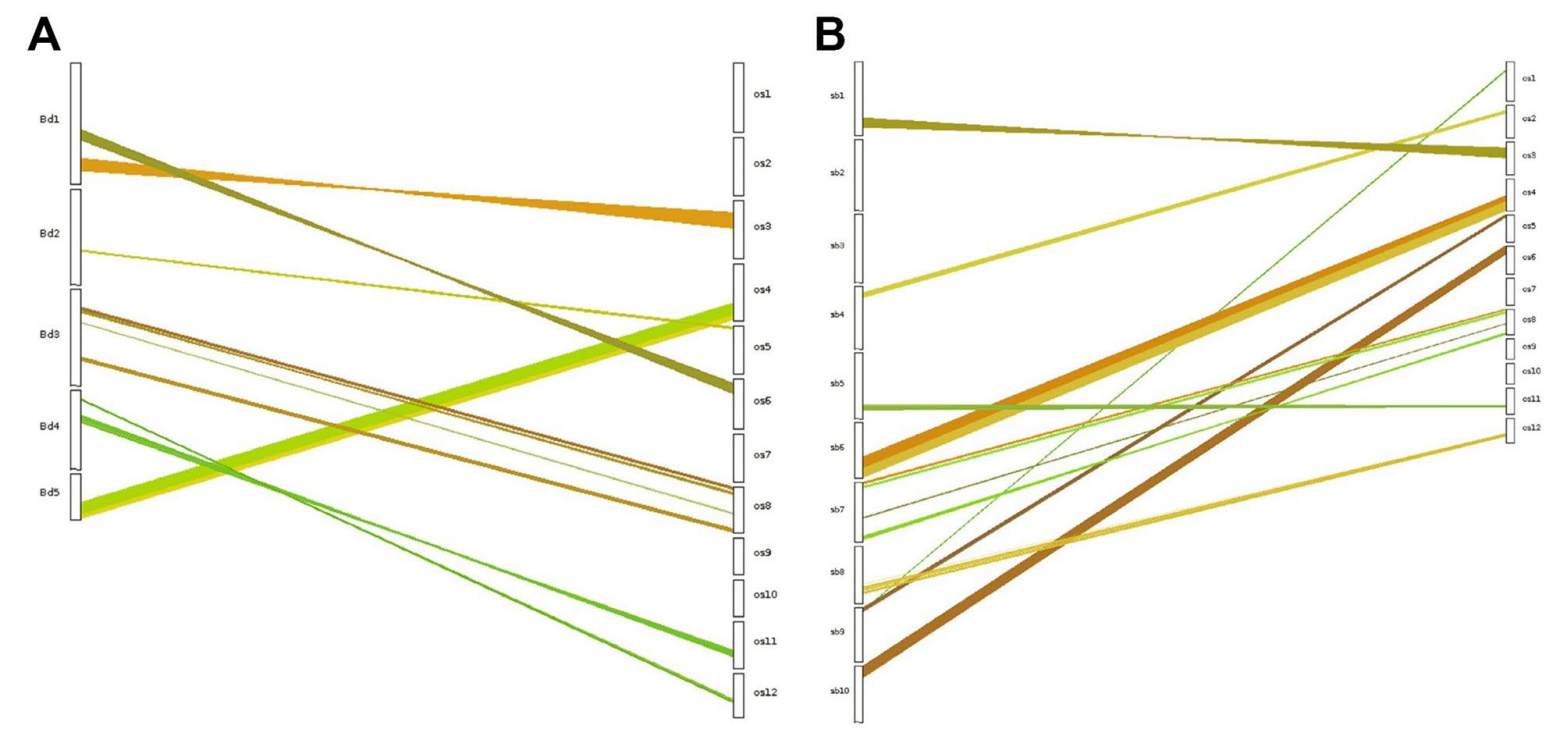
The synteny analysis of *DUF247* genes was conducted between rice and two representative plant species. **A**, synteny analysis was performed between rice and brachypodium. The colored lines represent collinear blocks of *DUF247* gene pairs between the rice and brachypodium genomes. **B**, The synteny analysis was performed between rice and sorghum. Likewise, the colored lines indicate collinear blocks of *DUF247* gene pairs between the rice and sorghum genomes. The species are denoted as follows: “Bd” represents brachypodium, “Os” represents rice, and “Sb” represents sorghum

To gain further insight into the phylogenetic mechanisms of the *OsDUF247* family, we constructed two comparative syntenic maps of *japonica* rice with two smaller genomic Gramineae species, including brachypodium and sorghum (Fig. 5 and Supplementary Table 7). Twelve and thirteen *OsDUF247* genes showed a syntenic relationship with those in brachypodium and sorghum, respectively. All genes were associated with their own syntenic gene pairs or syntenic blocks between rice and the other two species. Interestingly, 11 collinear pairs were identified between rice and the other two species, indicating that these orthologous pairs may have existed before their ancestral divergence. These collinear pairs were in the same phylogenetic groups, such as *LOC_Os01g19610.1-Sobic.009G030701.1* and *LOC_Os02g15430.1-Sobic.004G112400.1*, which both belonged to clade IA (Fig. 1B). The *Ka/Ks* ratios of these collinear pairs were also calculated, and the majority of the orthologous *DUF247* gene pairs had a *Ka/Ks* ratio < 1, suggesting that the *OsDUF247* gene family likely experienced strong purifying selective pressure during evolution. However, there were also two collinear pairs (*LOC_Os01g19610.1-Sobic.009G030701.1* and *LOC_Os05g03972.1-Sobic.009G030701.1*) with a *Ka/Ks* ratio > 1, which suggested that these two pairs likely experienced strong positive selection during evolution (detailed information of collinear gene pairs is listed in Supplementary Table 7).

### The expression pattern of *DUF247* genes in rice

**Fig. 6:**
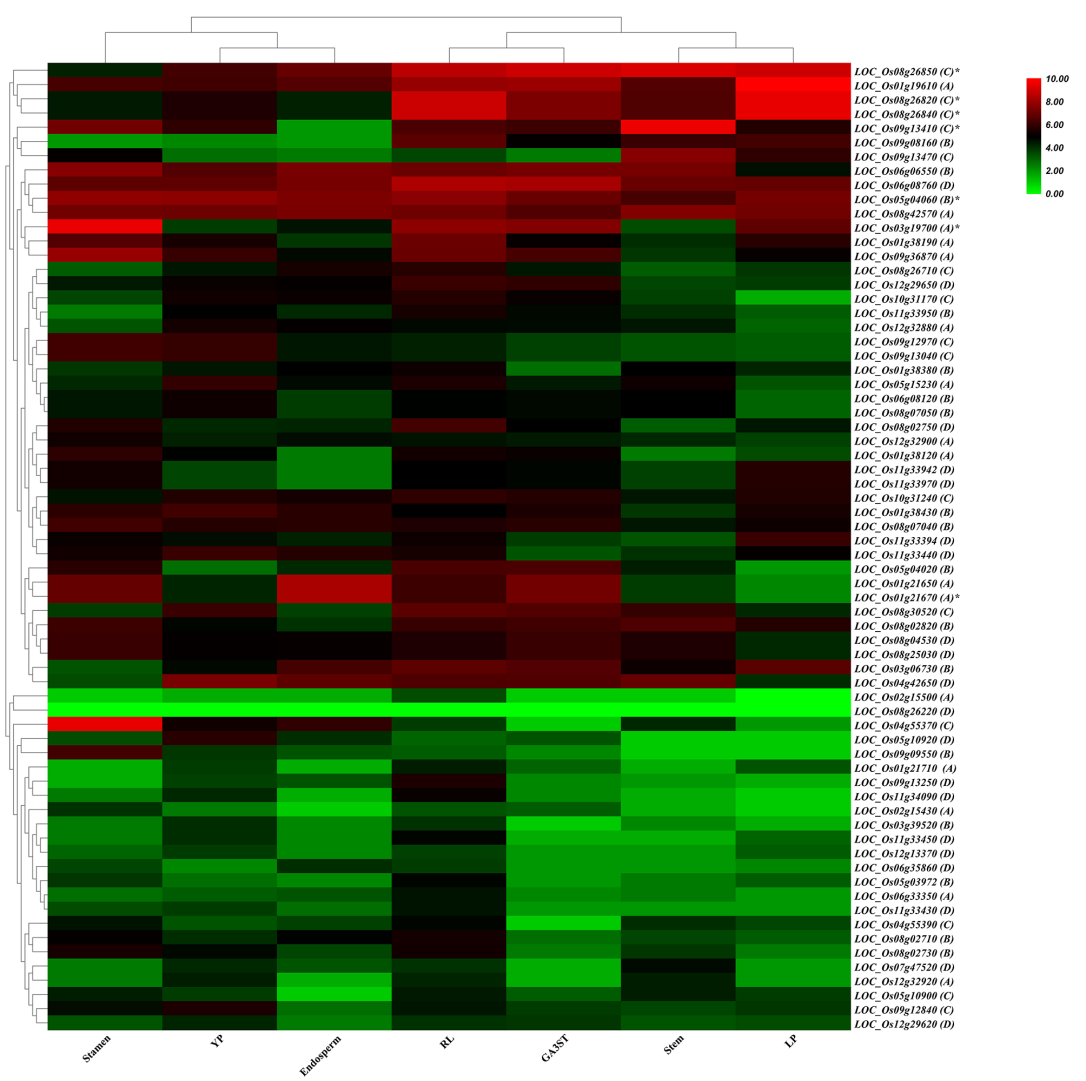
The expression heat map of *DUF247* genes in different tissues and organs. The heatmap was constructed using R. The clustering tree was constructed using the average linkage method hierarchical clustering. The detailed information of the expression data can be in Supplementary Table 8

**Fig. 7:**
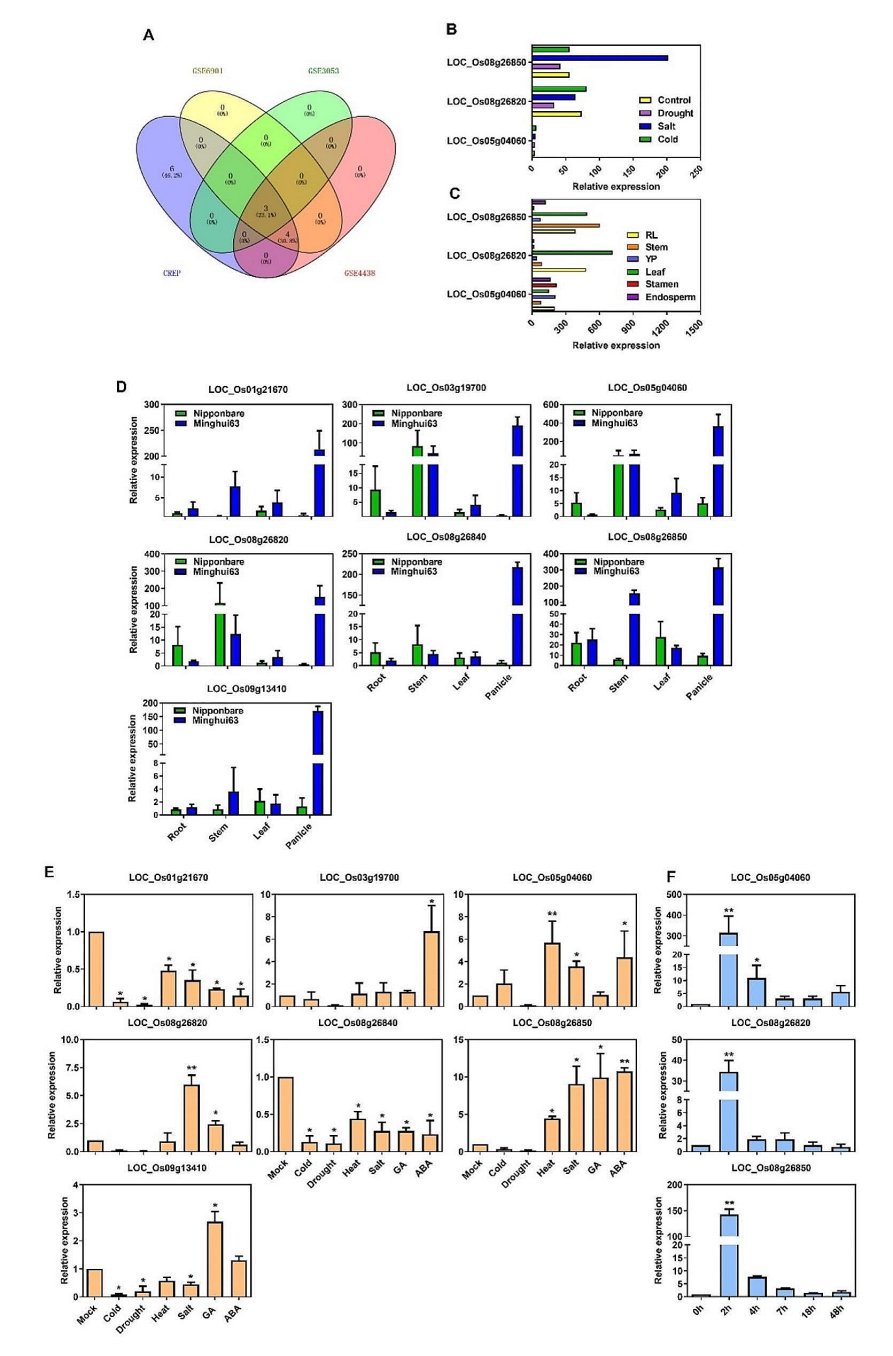
In-depth analysis of public expression microarray databases and qRT-PCR validation of the expression patterns of *DUF247* genes under normal and stress conditions. **A**, *DUF247* genes with relatively high expression were identified from four independent microarrays. **B**, Expression patterns of 3 common genes selected from the four databases under stress conditions. **C**, Expression patterns of 3 common genes selected from the four databases under normal conditions. **D**, Validation of the expression of 7 genes detected in four databases by qRT-PCR in different tissues. **E**, Validation of the transcriptional levels of 7 genes detected in four microarrays by qRT-PCR in various stress and hormone treatment. **F**, Validation of the transcriptional levels of 3 genes with high expression in four microarrays by qRT-PCR in fine salt stress treatment (200 mM NaCl) within 48 h. All the data mentioned were normalized using *OsActin* as the reference gene, and the normalization was performed based on three replicates with means ± SD, the differences between control and treatment were tested by LSD. Single asterisk indicates 0.01 < *P* < 0.05; double asterisks indicate *P* < 0.01. RL, root and leaf at three-leaf stage; stem, 5 days before heading; YP, young panicle at stage 5; leaf, young panicle at stage 3; Stamen, one day before flowering; Endosperm,7 days after pollination

To examine the expression patterns of *DUF247* genes in various tissues of Minghui63 (MH63) and Nipponbare (Nip) cultivars, we analyzed Affymetrix GeneChip transcriptome data for seedlings, roots, stems, leaves, young panicles, endosperm, and stamen at different developmental stages (Wang et al., 2010). The gene expression levels were quantified as fragments per kilobase per million (FPKM). The majority of *DUF247* genes had relatively low expression levels across the tested organs and tissues (Fig. 6, Supplementary Table 8). Among the 69 *DUF247* genes, *LOC_Os08g26220* and *LOC_Os02g15500* showed no or very low expression in all tested organs, indicating that they may be pseudogenes or have special temporal and spatial expression patterns not examined in this study. Around 44 genes showed constitutive expression across all detected tissues, while some genes exhibited tissue-specific expression patterns. For example, both *LOC_Os01g21650* and *LOC_Os01g21670* exhibited high expression in stamen and endosperm, but low expression in leaves. Overall, most *DUF247* genes from the same phylogenetic groups shared similar expression patterns. Only 13 genes expressed at higher levels (above 200) in at least one tissue (Supplementary Table 8). We also analyzed three other microarray databases (GSE6901, GSE3053, and GSE4438), which revealed that 6 (46.2%) of genes were present in GeneChips datasets, 3 (23.1%) of genes were common to all three microarrays, and 4 (30.8%) of genes were detected in two GSE databases (Fig. 7A).

To validate the transcriptome profiles of *DUF247* genes in different cultivars and tissues, we selected seven genes detected in the chips for qRT-PCR analysis. The results demonstrated that expression levels varied among different tissues (Fig. 7C), and the expression patterns were also different between the *indica* and *japonica* rice cultivars (Fig. 7D). For instance, *LOC_Os03g19700*, *LOC_Os05g04060*, and *LOC_Os08g26820* were highly expressed in the stem and lowly expressed in leaves in both the *indica* cultivar MH63 and the *japonica* cultivar Nip, while *LOC_Os01g21670* and *LOC_Os08g26850* were specifically highly expressed in the stem of MH63 but at a low level in Nip. Notably, all genes were expressed at specifically high levels in panicles in MH63 but at low levels in Nip indicating that some *DUF247* genes had low transcriptional activity, although several of them exhibited tissue-specific expression.

### Abiotic stress response of *DUF247* genes

**Fig. 8:**
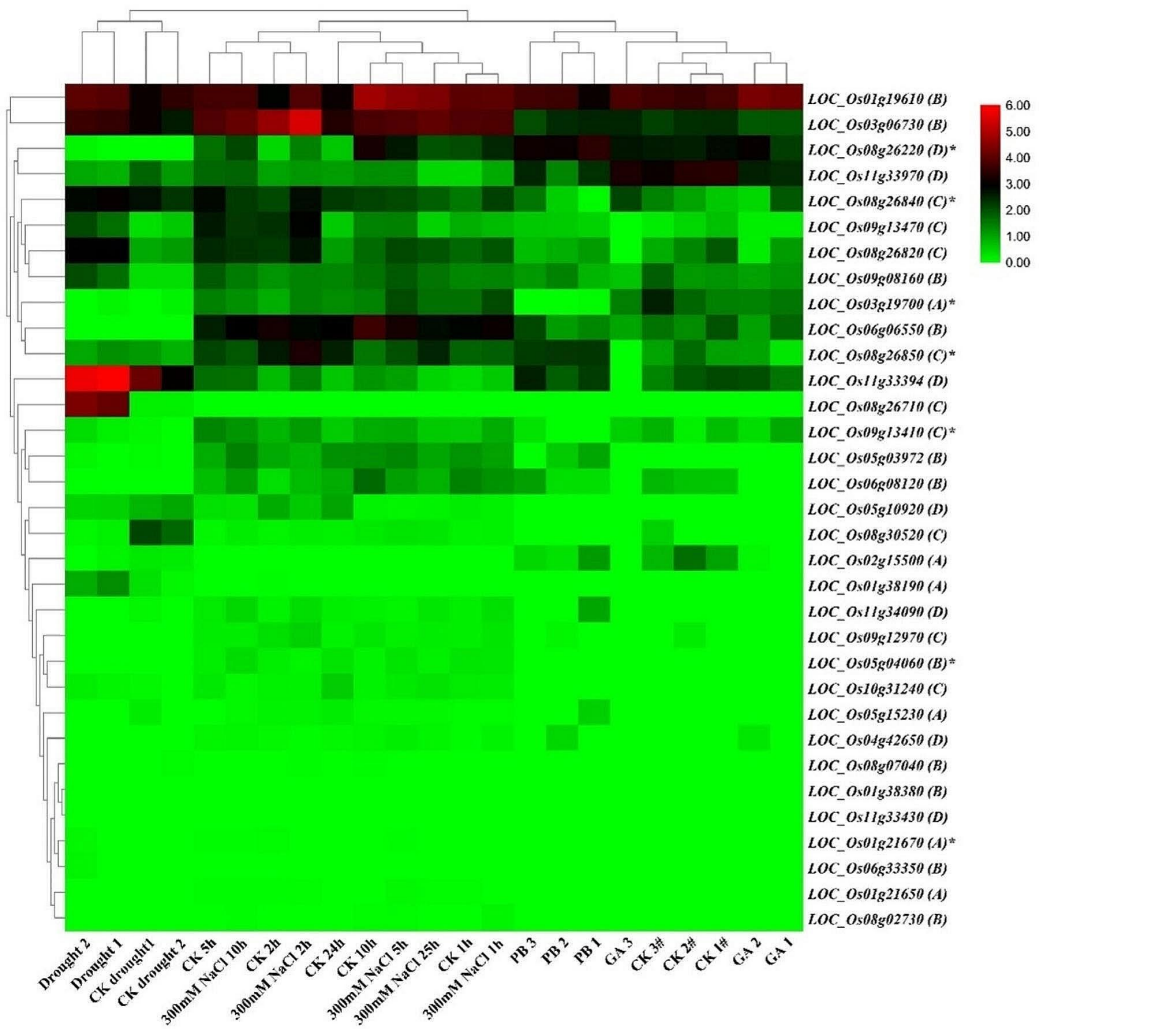
The expression profiles of detected *DUF247* genes under normal, salt stress, drought, GA, and paclobutrazol (PB) treatments. The detailed information of the expression profiles was listed in Supplementary Table 9

Our analysis of the microarray database revealed that *DUF247* were inducible by salt treatment in the *indica* cultivar Minghui63 (Fig. 7B). To further investigate their response to abiotic stresses, we searched the SRA database of rice for transcriptome data under salt, drought, gibberellins (GA), and paclobutrazol (PB) treatments (PRJEB4672, PRJNA408068, and PRJNA272723). We found that 26 *DUF247* genes were induced by these treatments (Fig. 8, Supplementary Table 9). Interestingly, some genes were significantly induced or repressed by multiple treatments. For example, *LOC_Os11g33394* was significantly repressed by drought and slightly repressed by GA, but remained insensitive to salt stress. Most genes were induced by salt stress, including the seven genes detected in the microarray database such as *LOC_Os03g19700*, *LOC_Os08g26220*, *LOC_Os08g26850*, and *LOC_Os08g26840*.

To gain further insight into the functions of these genes in the evolutionary process, we investigated the response of all seven genes to different abiotic treatments using qRT-PCR (Fig. 7E). Among them, *LOC_Os01g21670* and *LOC_Os08g26840* were repressed by multiple treatments, while *LOC_Os03g19700* was repressed by all treatments except ABA. *LOC_Os09g13410* showed repression under cold, drought, heat, and salt treatments, except GA and ABA treatments. Notably, three genes (*LOC_Os05g04060*, *LOC_Os08g26820*, and *LOC_Os08g26850*) detected in all gene chips were significantly induced by salt treatment. Additionally, *LOC_Os05g04060* was also induced by heat and ABA treatments, *LOC_Os08g26820* was induced by GA treatment, and *LOC_Os08g26850* was induced by heat, GA, and ABA treatments.

To further investigate the expression patterns of these three genes, qRT-PCR was performed under 200 mM NaCl treatment at different time points (Fig. 7F). The results showed that compared to the control, all three genes were significantly upregulated after two hours of salt treatment. Subsequently, their expression levels returned to normal within 48 h of treatment. It suggested that these genes are involved in the response to salt stress and may play a role in the rice plants to saline environments.

## Discussion

The *DUF247* gene family represents a large and recently discovered gene family in higher plants. Some members of this gene family have been found to play a crucial role in plant development, particularly in relation to self-incompatibility in grasses [22, 40]. However, the understanding of the overall role of the DUF247 family in development and stress response is still limited. We identified 69 *DUF247* genes from the rice genome, which were grouped into four clades based on their evolutionary relationships (Fig. 1). Moreover, the classification was corroborated by a conserved motif analysis, indicating that all identified DUF247 possess a common DUF247 domain and transmembrane motif (Fig. 2). This structural similarity aligns with findings observed in NtDUF868, ZoDUF668, and OsDUF568 families, as previously reported [18–20]. These findings suggest a potential role for these genes in responding to abiotic and biotic stresses [21].

In Gramineae species and dicot, the number of *DUF247* genes varies across different species (Supplementary Table 4). Interestingly, the expansion of the *DUF247* gene family in rice appears to be relatively faster compared to other grass species, despite its smaller genome size. It suggests that factors other than genome size are influencing the expansion of this gene family in rice. The clustering of some *DUF247* genes on chromosomes suggests the occurrence of duplication events within the gene family. Duplication events drive the distribution and expansion of genes in the genome, and similar observations have been made in other plant gene families, such as the NBS-LRR protein family and F-box protein family [35, 41, 42]. Phylogenetic tree analysis demonstrated that both Gramineae and dicotyledonous plants may share a common ancestor (Fig. 1B), similar to the DUF568 and DUF4228 family [16, 20].

Gene duplication events, including tandem, segmental, and transposition duplications, are essential in genome replication and contribute to the formation of gene families [38]. In the rice *DUF247* gene family, three gene pairs evolved from tandem duplication, while 18 segmental duplications, which play a major role in gene family expansion, were identified (Figs. 3 and 4). To examine the syntenic relationship between rice and other Gramineae, we analyzed the genomes of two species with smaller genome sizes, brachypodium and sorghum. The results revealed consistent collinearities between rice and these two species, indicating conservation among Gramineae.

Several *DUF* genes have been implicated in the response to drought and salt stress. *GmCBSDUF3* overexpression, containing DUF21 domain, enhanced drought and salt stress tolerance in Arabidopsis [43]. The *AhDGR2* gene in *Amaranthus hypochondriacus*, encoding a DUF642-domain-containing protein, when overexpressed in Arabidopsis, led to increased sensitivity to NaCl treatment [44]. Expression profiling analysis revealed that certain *TaDUF966* genes in wheat (*Triticum aestivum* L.) were induced by salt stress, and *TaDUF966-9B* was further confirmed to play a role in salt stress through virus-induced gene silencing (VIGS) assay [45]. OsSIDP366, containing DUF1644, positively regulates drought and salt stress responses in rice by regulating PBs/SGs [46]. On the other hand, *OsDSR2*, encoding a DUF966-domain-containing protein, negatively [15]. Analyses of microarray databases showed that the expression levels of most *DUF247* genes were generally low in all investigated tissues or organs, with some genes displaying tissue-specific expression (Fig. 7A), suggesting these genes may represent major functional members of the *DUF247* family in response to abiotic stress. Further analysis of the expression patterns of *DUF247* genes under various abiotic stresses demonstrated their regulation by multiple stresses, particularly salt stress (Fig. 7E and F). Further investigation is needed to gain a deeper understanding of the molecular mechanism underlying the regulation of salt stress by *DUF247* genes.

The presence of the *DUF247* gene family in the rice genome, as well as its response to various abiotic and biotic stresses, suggests its involvement in stress response and reproductive development. These findings provide valuable insights into the potential functional roles of *DUF247* genes. The comprehensive analyses conducted in this study will be beneficial for selecting specific *DUF247* genes for further functional characterization and genetic improvement of agronomic traits. Moreover, these findings can contribute to enhancing the environmental resistance of new rice cultivars.

## Conclusions

In this study, a total of 69 protein sequence entries with a predicted DUF247 domain were identified and were conducted to estimate evolutionary analysis in rice. Gene structure and conserve motif analysis indicated the conservation and dispersal of DUF247 family during evolution. Furthermore, expression profiling and protein interaction predictions suggest that rice *DUF247* genes might be involved in plant resistance to environmental stresses. These findings lay the foundation for functional characterizations of *DUF247* genes to unravel their exact role in rice cultivars.

### Electronic supplementary material

Below is the link to the electronic supplementary material.

Supplementary Material 1

Supplementary Material 2

Supplementary Material 3

Supplementary Material 4

Supplementary Material 5

Supplementary Material 6

Supplementary Material 7

Supplementary Material 8

Supplementary Material 9

## Data Availability

All data generated or analysed during this study are included in this published article.

## Abbreviations

DUF247: Domain of unknown function 247
GA: Gibberellin
PB: Paclobutrazol
HMM: Hidden Markov model
Mw: Molecular weight
pI: Isoelectric point
